# Geographical and climatic risk factors for COVID-19 in southwest Iran during the 2020–2021 epidemic

**DOI:** 10.1371/journal.pone.0336595

**Published:** 2025-11-12

**Authors:** Koorosh Nikaein, Zahra Kanannejad, Mohammad Amin Ghatee

**Affiliations:** 1 Student Research Committee, Yasuj University of Medical Sciences, Yasuj, Iran; 2 Allergy Research Center, Shiraz University of Medical Sciences, Shiraz, Iran; 3 Professor Alborzi Clinical Microbiology Research Center, Shiraz University of Medical Sciences, Shiraz, Iran; 4 Department of Microbiology, School of Medicine, Yasuj University of Medical Sciences, Yasuj, Iran; Satyawati College, University of Delhi, INDIA

## Abstract

The COVID-19 pandemic has had a devastating impact worldwide, with Iran being one of the hardest-hit countries in the Middle East. Understanding the factors that influence the spread of the virus is crucial for developing effective mitigation strategies. This study aims to investigate the geographical and climatic risk factors associated with COVID-19 incidence in the Kohgiluyeh and Boyer-Ahmad Province of southwest Iran during the 2020−2021 epidemic period. The study involved mapping the residential addresses of 15,585 patients with COVID-19 during 2020–2021. Geographical Information System (GIS) evaluated the effects of geographical and climatic determinants, including temperature, rainfall, humidity, evaporation, elevation, slope, and land cover, on COVID-19 occurrence. The data were analyzed using univariate and multivariate binary logistic regression. In the univariate model, significant climatic factors affecting COVID-19 susceptibility included elevation (p < 0.001, OR=0.617), evaporation (p < 0.001, OR=0.635), dusty days (p < 0.001, OR=1.050), humidity (p = 0.005, OR=1.013), and rainfall (p = 0.032, OR=0.998). Additionally, urban areas (p < 0.001, OR=65), irrigated farms (p < 0.001, OR=5.723), dry farms (p < 0.001, OR=3.101), thin forests (p = 0.009, OR=2.975), and thin rangeland (p = 0.030, OR=2.571) demonstrated the highest impact on the disease distribution. In the multivariate analysis, urban areas (p < 0.001 and OR=47.123), irrigated farms (p < 0.001, OR=4.510), dry farms (p = 0.006, OR=3.002), evaporation (p < 0.001, OR=0.999), and elevation (p < 0.001, OR=0.999) were found to be the main factors related to COVID-19 occurrence. Based on the study results, individuals living in urban areas, irrigated and dry farms, as well as in regions with lower elevation and lower evaporation, have a higher risk of contracting COVID-19.

## 1. Introduction

The Coronavirus Disease 2019 (COVID-19) pandemic has had a devastating global impact, with significant variations in incidence and severity across different regions. Understanding the factors that contribute to these geographical differences is crucial for informing public health interventions and policies. Previous studies have suggested that environmental, climatic, and population-level characteristics may play an important role in the spread and susceptibility of SARS-CoV-2 [1,2].

COVID-19 transmission primarily occurs through close contact between an infected person and an uninfected person through exposure to aerosols or droplets carrying the infectious SARS-CoV-2 virus. Contact with contaminated surfaces or objects can also transmit COVID-19, but the risk of infection is much lower [3]. This implies that the presence, survival, and infectivity of the virus in the air and on object surfaces could be affected by environmental conditions, such as temperature, humidity, precipitation, air quality, and certain geographical features [4–6].

Research indicates that temperature has a significant effect on the transmission of COVID-19, with studies showing an inverse relationship between temperature and the daily number of infections [7]. Humidity levels are also critical in understanding COVID-19 transmission; lower absolute humidity environments have been correlated with enhanced survival rates of the virus [8]. The influence of precipitation on COVID-19 transmission appears more inconsistent than that of temperature and humidity. Its effects can vary significantly depending on the geographical context, suggesting that while some regions might see enhanced virus spread associated with rainfall, others may not exhibit any such correlation [9].

Geographical features, such as population density, also have notable implications for COVID-19 incidence. Urban areas tend to experience higher transmission rates due to the increased potential for close human contact. Studies suggest that initial urban density fosters rapid virus spread, but this relationship may change as social distancing measures are adopted or as public compliance with such measures varies [10].

Geographic Information Systems [GIS] is a powerful tool for studying the relationship between geographic, climatic, and environmental factors and the spread of diseases using mapping, spatial analysis, and correlation studies [11]. GIS enables the identification of areas with a high risk of disease and the analysis of the connection between geographical characteristics and disease prevalence through the creation of thematic maps [12–14]. Important geographical and climatic variables, including temperature, UV radiation, wind speed, humidity, air quality, and land cover, have been identified as the primary drivers of COVID-19 transmission on a global scale using GIS methods [15,16]. In Iran, most GIS-based studies on COVID-19 have focused on population density, while limited studies considered geographical and climatic features [17,18]. The current study employed GIS to comprehensively investigate the influence of key geographical and climatic factors on the occurrence of COVID-19 in the Kohgiluyeh and Boyer-Ahmad Province of southwest Iran. This province has unique conditions, as it encompasses a range of climatic zones: from hot and semi-arid areas in the south and southwest to cold and wet regions in the north and east of the province. This diversity makes the province an ideal location to assess COVID-19 distribution across different climatic conditions within a relatively small area. The variables investigated were included humidity, temperature, evaporation, dusty days, precipitation, wind speed, elevation, slope, and land cover. By analyzing the spatial relationships between these environmental characteristics and COVID-19 incidence, the study aimed to identify high-risk areas where the disease is more prevalent and environmental conditions are more conducive to its spread. The insights gained from this GIS-based analysis can inform the development of more targeted and effective strategies for controlling the COVID-19 pandemic in the region.

## 2. Methods

### 2.1. Study area

**Fig 1:**
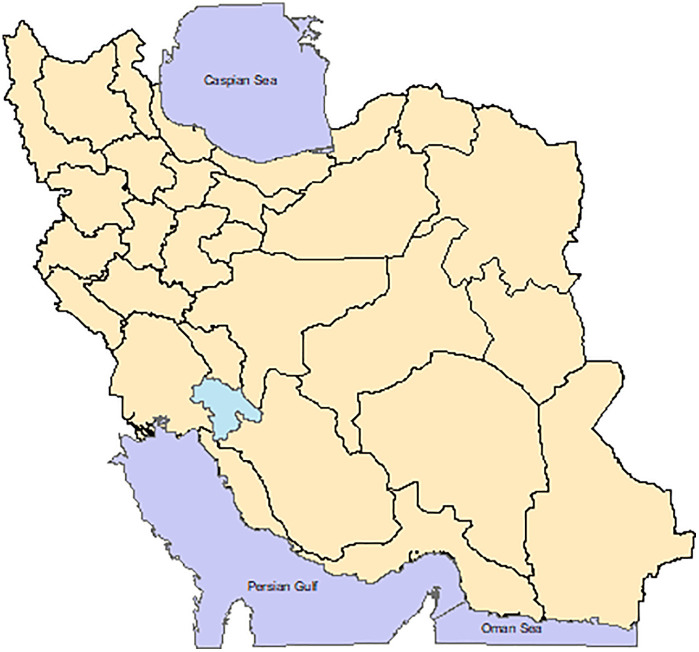
Location of the study. Kohgiluyeh and Boyer-Ahmad province as study area in southwest Iran.

The study was done in Kohgiluyeh and Boyer-Ahmad Province with geographical location 30.67°N and 51.60°E. It is located in the southwest of Iran and its capital is Yasuj. The province covers an area of 15,563 square kilometers. It encompasses regions situated in some of the highest elevations in the central portion of the Zagros Mountain range including Dena Peak, which ranks as the seventh highest peak in Iran, with an elevation of 4,409 meters. The research region offers a wide range of climates and vegetation, consisting around 2,000 different plant species (Fig 1).

### 2.2. Patients

The data of 15,585 patients confirmed as COVID-19 by real-time PCR during 2020–2021 were retrieved from the Provincial Health Center of Kohgyloyeh and Boyerahmad province. Considering the high number of patients, all patients from the middle months of four seasons that includes 8 months over two years were included. The dwelling addresses of patients were pointed on the political map of the province including villages and cities. This study was approved by the Ethics Committee of Yasuj University of Medical Sciences (Ethics Approval No: IR.YUMS.REC.1401.121). The current project is a retrospective study, and patients’ data were retrieved anonymously from the Health Center records in accordance with ethical rules and regulations set by the ethics committee. No clinical samples were taken from patients. Consent to participate was deemed unnecessary according to the Ethic Committee of Vice president for Research and Technology, Yasuj University of Medical Sciences. This study was initiated after approval by the ethics committee on February 10, 2023, and was finalized three months later.

### 2.3. Geographical and meteorological data

All meteorological data for the two-year period of 2020–2021 including temperature, rainfall, humidity, evaporation, and wind speed were collected from the Kohgiluyeh and BoyerAhmad Province Weather Bureau. This information was collected daily. The mean annual values for each factor were then calculated and recorded as mean annual temperature (MAT), maximum mean annual temperature (MaxMAT), minimum mean annual temperature (MinMAT), mean annual evaporation (MAE), mean annual dusty days (MADD), mean annual humidity (MAH), and mean annual rainfall (MAR).

The Department of Natural Resources in Kohgiluyeh and Boyer-Ahmad Province acquired geographical information, including a digital elevation model raster layer and a land cover vector layer. The slope raster layer was derived from the digital elevation model (DEM) map using the spatial analyst tool to determine the highest possible gradients between each cell and its neighboring ones.

### 2.4. Geospatial analysis

The isohydral and isohumid raster layers were created with the Kriging interpolation technique, while the isothermal, isoevaporation, and isowind speed layers were developed using the tension-based Spline interpolation model after evaluating the various interpolation methods. The resolution grid used for all layers was 1 × 1 km. The point layers indicating villages and towns within the analyzed areas were generated from the raster layers. Subsequently, the geometric intersections of the result layer and the land cover vector layer were determined by the Identity instrument, leading to the development of the final layer. The final layer contained the geographical and climatic data obtained from the overlapped raster and vector layers at every location. The connection between geographical and climatic parameters and COVID-19 was assessed by examining the geographic distribution of patients living in Kohgiluyeh and Boyer-Ahmad Province. Data related to residential areas, including both cities and villages with reported COVID-19 cases and those without reported cases, were extracted from the final layer of provincial city and village points. The information was then analyzed by both, univariate and multivariate logistic regression models. Prior to multivariate analysis, independent variables were checked for collinearity by pairwise correlation and variance inflation factor (VIF). The statistical assessments were performed using SPSS 21 version.

## 3. Result

### 3.1. Geographical and climatic distribution of regions influenced by COVID-19

**Fig 2:**
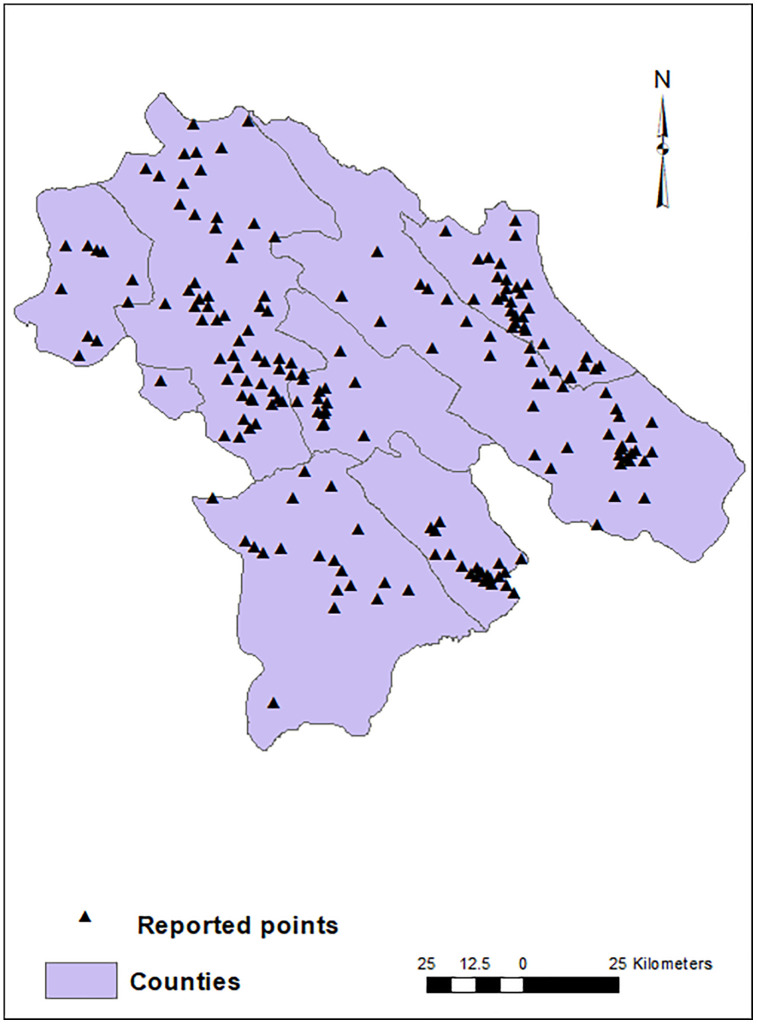
Geographical distribution of infected points in Kohgiluyeh and Boyer-Ahmad province.

**Fig 3:**
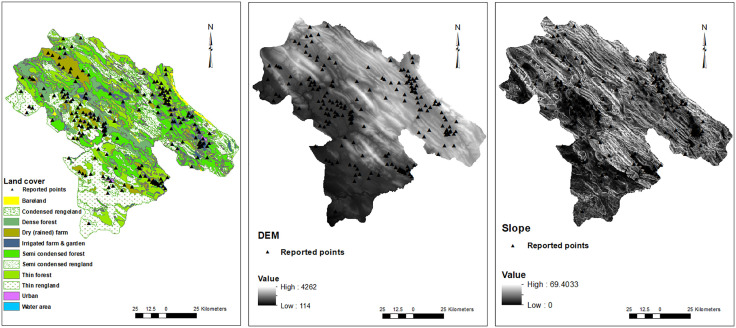
Geographical factors and distribution of COVID-19 cases. The triangle symbols on the maps indicate the locations of confirmed COVID-19 cases in the region. DEM (digital elevation model).

**Fig 4:**
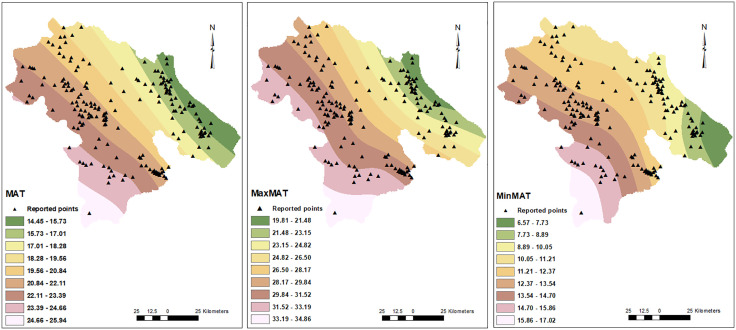
Mean temperature and distribution of COVID-19 cases. The locations of confirmed COVID-19 cases are shown by triangle symbols overlaid on the maps, allowing visualization of how the infections are spatially distributed within the geographical context according to temperature. Mean annual temperature (MAT), maximum (max), minimum (min).

**Fig 5:**
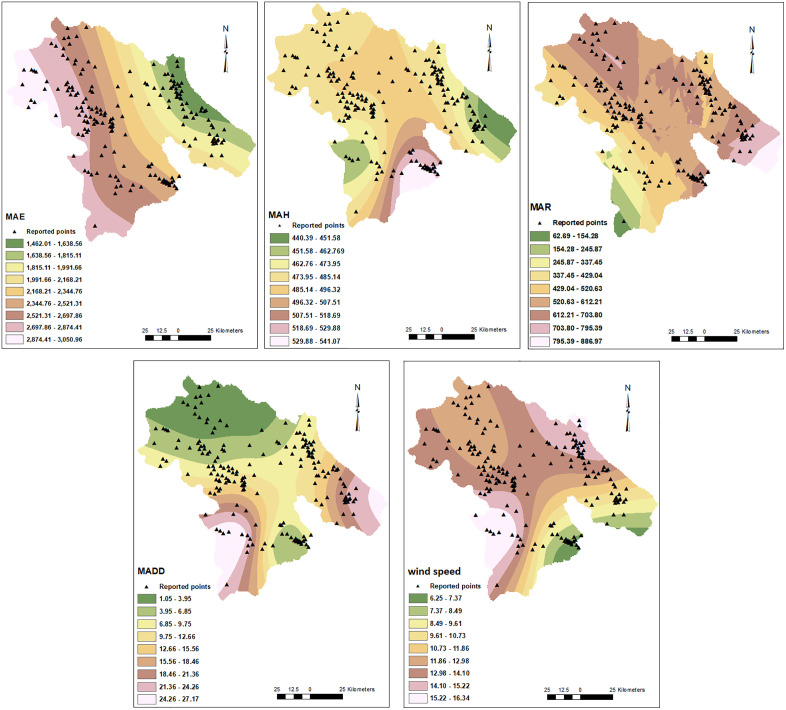
MAR, MAH, MAE, MADD, and wind speed raster models. Points with COVID-19 were shown by triangle symbol. Mean annual rainfall (MAR), mean annual humidity (MAH), mean annual evaporation (MAE), mean annual dusty days (MADD).

In this study, the dwelling addresses of 15,585 out of 98,464 patients whose data had been recorded in the Provincial Health Center were included. These patients were reported from 190 points among all 2213 points in this province. The geographical distribution of these COVID-19 patients in Kohgiluyeh and Boyer-Ahmad province is shown in Fig 2. These points located in varied geographical and climatic areas of the province as illustrated in Figs 3–5.

### 3.2. Univariate logistic regression

#### 3.2.1. Meteorological factors.

Several meteorological factors were identified as significant contributors to COVID-19 occurrence, including MAE, MADD, MAH and MAR. An increase in MAE (OR=0.999, 95% CI = 0.999–1.000) and MAR (OR=0.998, 95% CI = 0.997–1.000) by one millimeter reduces the chance of COVID-19 by 0.1% and 0.2% respectively. On the other hand, an increase in MADD (OR=1.05, 95% CI = 1.028–1.073) by one day and MAH (OR=1.013, 95% CI = 1.004–1.022) by one percentage increases the chance of COVID-19 by 5% and 1.3%, respectively (Table 1). However, other climatic variables were not found to be significant factors affecting disease occurrence.

**Table 1:** Effects of climatic variables on COVID-19 occurrence.

Variable	P- value	Odd’s ratio	CI
MAT	0.482	0.977	0.917-1.042
min MAT	0.933	1.004	0.923-1.091
max MAT	0.259	0.973	0.928-1.020
MAR	**0.032**	0.998	0.997-1.000
MAH	**0.005**	1.013	1.004-1.022
MAE	**0.020**	0.999	0.999-1.000
Wind speed MADD	0.855 **< 0.001**	1.008 1.050	0.930-1.092 1.028-1.073

Mean annual temperature (MAT), maximum (max), minimum (min), mean annual rainfall (MAR), mean annual humidity (MAH), mean annual evaporation (MAE), and mean annual dusty days (MADD).

#### 3.2.2. Geographical factors.

In this study, urban setting, irrigated farms, dry (rainfed) farms, thin forests, thin rangelands, and DEM were identified as the main geographical determinants of COVID-19 occurrence. The likelihood of contracting COVID-19 was found to be increased by 65 (OR = 65, 95% CI = 19.644–215.82), 5.723 (OR = 5.723, 95% CI = 2.690–12.176), 3.101 (OR = 3.101, 95% CI = 1.427–6.737), 2.975 (OR = 2.975, 95% CI = 1.311–6.754), and 2.571 times (OR = 2.571, 95% CI = 1.096–6.035) in residents living in urban settings, irrigated farms, dry (rainfed) farms, thin forests, and thin rangelands areas, respectively. On the other hand, the risk of the disease decreased by 0.001% by increasing each meter in elevation (OR = 0.999, 95% CI = 0.999–1000) (Table 2).

**Table 2:** Effects of geographical variables on COVID-19 occurrence.

Variable	P- value	Odd’s ratio	CI
Land covers			
Bare land	0.999	0.000	0.000
Thin rangeland	**0.03**	2.571	1.096-6.035
Semi-condensed rangeland	0.123	2.031	0.825-5-001
Condensed rangeland	.983	1.017	0.210-4.915
Dry farm	**0.004**	3.101	1.427-6.737
Irrigated farm	**< 0.001**	5.723	2.690-12.176
Water area	1.000	0.000	0.000
Urban	**< 0.001**	65.000	19.644-215.082
Thin forest	**0.009**	2.975	1.311-6.754
Semi-condensed forest	0.731	0.847	0.330-2.178
Dense forest	< 0.001		
DEM	**< 0.001**	0.999	0.999-1.000
Slope	0.982	1.000	0.999-1.001

Digital elevation model (DEM).

### 3.3. Multivariate logistic regression model

The multivariate analysis employed the forward stepwise method in multivariate regression analysis to determine the significant factors in the univariate regression model. For this, variables were added step by step based on the significance of the score statistic. Only three factors were found to be associated with increased COVID-19 occurrence: residing in an urban area (OR = 47.123, 95% CI = 13.838–160.446), having an irrigated farm (OR = 4.510, 95% CI = 2.025–10.043), and having a dry farm (OR = 3.002, 95% CI = 1.375–6.557). Conversely, there was a decreasing trend found for DEM (OR = 0.999, 95% CI = 0.998–0.999) and MAE (OR = 0.999, 95% CI = 0.998–0.999). No additional geographical and climatic parameters were identified to be significant variables (Table 3).

**Table 3:** Forward stepwise multivariate analysis of the effect of geographical and climatic factors on COVID-19 in the Kohgiluyeh and Boyer-Ahmad Province.

Variable	P- value	Odd’s ratio	CI
MAE	**0.012**	0.999	0.998-0.999
DEM	**<0.001**	0.999	0.998-0.999
Bare land	0.999	0.000	0.000
Thin rangeland	0.189	1.787	0.752- 4.246
Semi-condensed rangeland	0.130	2.015	0.813- 4.989
Condensed rangeland	0.413	1.966	0.390- 9.907
Dry farm	**0.006**	3.002	1.375- 6.557
Irrigated farm	**< 0.001**	4.510	2.025- 10.043
Water area	1.000	0.000	0.000
Urban	**< 0.001**	47.123	13.838-160.466
Thin forest	0.061	2.283	0.962-5.415
Semi-condensed forest	0.692	0.819	0.305-2.198
Dense forest (constant)	0.163	5.198	

Mean annual temperature (MAT), Mean annual rainfall (MAR), Digital elevation model (DEM).

## 4. Discussion

It is widely accepted that changes in environmental and weather conditions can affect the transmission and distribution of various diseases, and the researchers aimed to investigate the specific impact of these factors on the spread of COVID-19 in the region. In the current study, urban residential areas, irrigated farms, dry farms, evaporation, and altitude were the most important geoclimatic determinants of COVID-19, respectively. Moreover, COVID-19 incidence was also separately affected by humidity, rainfall, frequency of dusty days and thin rangeland and forest when their effect was evaluated independently.

### 4.1. Geographical factors

The urban setting, which according to satellite images includes cities and large villages was the most effective environmental factor in our study. The hypothesis that densely populated regions such urban areas would become epicenters of virus transmission prompted a surge of academic research on the topic. Several studies suggested urban life led to faster spread of disease in the USA, Japan and India and China [19–23]. Nguimkeu and Tadadjeu (2021) conducted a study analyzing demographic and geographic factors in 182 countries. They discovered that population density, urbanization rate, and the proportion of people aged 65 and over were the key factors in COVID-19 distribution [24]. Overall, it has been shown that living in urban residential areas increases the risk of contracting the virus due to close contact between people [25]. In addition, urban settings are considered socioeconomic hubs for surrounding and even distant regions, leading to more interpersonal contact, which may account for increased disease prevalence in these areas.

On the other hand, some studies have failed to find a correlation between population density and the rate of COVID-19 transmission. In a study conducted in New York City, population density was not a risk factor, whereas the socioeconomic level of citizens was strongly associated with the disease [26]. Boterman (2020) found no evidence that urban density significantly influenced COVID-19 outbreak prevalence in Netherland. They argued this contrasts with other European studies (e.g., Italy and Belgium) and suggested that because the Netherlands is highly urbanized with high population densities even in rural areas coupled with intensive agro-industries, the difference in COVID-19 prevalence between rural and urban areas was insignificant [27].

Irrigated and dry farmland were other land covers associated with COVID-19 incidence in this study. In a study by Páez-Osuna et.al, it was found that irrigated and dry farm were more susceptible to the spread of COVID-19 due to increased dust originating from these regions [28]. Dusty places are more suitable for respiratory illnesses like asthma [29]. This result also supports the significantly positive association between MADD and COVID-19 incidence in our study that will be discussed in the climatic factor part. When the wind moves pathogens that cause respiratory infections become suspended in the air and adhere to dust particles. The particles pass the body’s inherent filtration processes, such as the nose, hair, and throat mucus, and aggregate in the lungs, potentially leading to the development of illnesses. Endotoxins, gram-negative bacteria, fungi, and β-d-(1-3)-glucan are types of biological agents that can be found in organic dust. Overexposure to these agents can lead to the development of organic dust toxic syndrome, upper respiratory tract infections, and extrinsic allergic alveolitis (EAA) [30].

Elevation was a significant factor associated with a slight decrease in COVID-19 risk in our study. Physiological factors can affect the pathogenicity of COVID-19 at high altitudes [31]. Research has been shown that high-altitude natives have certain physiological benefits like enhanced hypoxic ventilatory response, higher levels of oxygen-carrying hemoglobin, and increased Vitamin D production due to strong sunlight exposure, along with lower incidence of conditions like lung infections, obesity, etc. [32]. These factors combined may explain the enhanced resilience to SARS-CoV-2 infection seen in high-altitude natives [31]. Additionally, lower air density and greater distance between air molecules at higher altitudes may reduce the spread of airborne viruses compared to sea-level areas [33]. Christian Arias-Reyes et al. analyzed the epidemiological data of COVID-19 in Tibet and high-altitude regions of Bolivia and Ecuador, and compared it to data from lowland areas. Their aim was to test the hypothesis that individuals living at high altitudes (+2,500 m above sea level) are less likely to develop severe adverse effects from acute SARS-CoV-2 virus infection. They proposed that physiological acclimatization or adaptation to the hypoxic environment at high altitudes may offer protection against the severe impact of acute SARS-CoV-2 virus infection [34].

In the univariate analysis conducted in this study, thin rangelands and thin forests were identified as significant land cover factors affecting COVID-19 incidence. To the best of our knowledge, no studies have directly evaluated the effect of thin rangeland and thin forest on COVID-19 transmission. However some studies have reported increased visitation to urban forests and other green spaces during the COVID-19 pandemic, as these areas served as vital venues for social interaction and recreation [35]. Additionally, research has highlighted the role of forests in reducing stress, anxiety, and cortisol levels during the pandemic, which may explain the heightened public attraction to these areas and increased human to human exposure [36]. A study in Nepal further revealed that many individuals returned to their rural villages after losing urban jobs during the pandemic, leading to increased reliance on nearby forest resources [37]. This surge in forest and rangeland use around residential areas resulting in greater human contacts may also help explain the observed patterns of COVID-19 distribution in the present study.

### 4.2. Climatic factors

Evaporation is one of the main factors that could slightly decrease disease occurrence in this study, likely by reducing virus survival and decreasing transmission via virus-containing droplets. The rate of evaporation is influenced by the thermal conductivity and thickness of the substrate, as well as the relative humidity of the air. The virus can survive longer in environments with low evaporation compared to dry conditions with high evaporation [38]. On the other hand, increasing the evaporation in the environment reduces the spread of the disease by making the contaminated droplets smaller and easier to manage [39]. The current study, along with prior research, indicates that increased evaporation rates could result in a reduction in COVID-19 cases [38,40]. Dry weather leads to rapid evaporation of the moisture layer around the virus. The removal of moisture from these external parts may be a natural process, which can reduce the virus’s infectivity [41,42].

Airborne particles have been identified to have both long-term and short-term effects on human health, especially in relation to cardiopulmonary and respiratory illnesses [43]. Several epidemiologists have observed that mortality rates are higher on days with greater amounts of particulate matter (PM) compared to days with lower levels of dust [44]. Dust storms can transport bioaerosols over vast distances, impacting ecosystems and populations located downwind. Dust and sandstorm events have the potential to introduce a diverse range of foreign microorganisms into the global system. The bacteria present in dust are extremely persistent and show remarkable resistance to several environmental pressures, including dehydration, harsh temperatures, high salt levels, and UV radiation exposure [45,46]. Furthermore, the existence of particulate matter can trigger the activation of transmembrane serine protease 2 (TMPRSS2) and angiotensin-converting enzyme 2 (ACE-2), leading to an increase in the number of SARS-CoV-2 binding sites and infection efficiency [47,48]. In the present study, a clear association between the number of dusty days and COVID-19 incidence has been identified. Many studies have also found a direct relation between air quality and SARS-CoV-2 infection. A study conducted in Istanbul, Turkey examined the correlation between air quality indicators and the daily number of COVID-19 cases. The study revealed that there is a connection between air quality and the transmission of the disease within the community [49]. A study conducted in the state of New Jersey, United States, detected a direct relationship between the number of newly reported COVID-19 cases and the air quality index [50]. In April 2020, the Middle East dust phenomenon penetrated Khuzestan province in southwest Iran, leading to a significant increase in the daily rate of the disease. The number of new registered cases tripled within 7 days of the phenomenon, indicating an abnormal rise. A study analyzing the data revealed a direct association between the dust and the frequency of positive COVID-19 cases in Khuzestan, southwest Iran [51].

Rainfall was identified as significant factors slightly affecting COVID-19 incidence in current study. Increased rainfall was associated with reduced disease rates. Previous studies have also found that rainfall was negatively related to COVID-19 incidence [52–55]. Chien and Chen observed a noteworthy inverse correlation between rainfall and the occurrence of COVID-19 in the USA. They found that the number of daily cases increased when rainfall ranged from 1.27 to 1.74 inches, but decreased when rainfall exceeded 1.77 inches [54]. Similarly, Menebo reported a substantial inverse relationship between daily precipitation amounts and COVID-19 in Oslo, Norway [55]. This could be due to the fact that people prefer to “stay at home” on rainy days, which reduces interactions between people [56]. Some researchers suggest that higher rainfall level may help reduce the spread of the virus by washing away viral particles in the air. In addition, higher rainfall may lead to fewer dusty days and lower disease incidence [57]. However, the search results indicate that the evidence on the correlation between rainfall and COVID-19 incidence remains and inconclusive. No significant correlation was found between rainfall and COVID-19 in studies conducted in New York, USA, Jakarta, Indonesia, or NSW, Australia [58–60]. In contrast to our results, certain research have discovered a direct correlation between rainfall and the incidence of COVID-19 [4,61,62], potentially related to the weakening of infected persons’ respiratory and immune systems or increased relative humidity after heavy rainfall [63].

Humidity is a key climatic factor associated with COVID-19 incidence in most studies. Prior research has provided evidence to support the epidemiological concept that dry conditions with low absolute humidity promote the survival and spread of viral infections transmitted through droplets. On the other hand, humid environments with high absolute humidity experience reduced transmission of viruses, such as influenza. However, the impact of absolute humidity on the transmission of COVID-19 remains uncertain. The relationship between humidity and COVID- 19 has been found to be positive in the current study. These results are consistent with other studies [64–66]. Wei Luo et al. analyzed the variation in the basic reproductive numbers of COVID-19 at the provincial level in China. They found that changes in weather, specifically the rise in temperature and humidity during spring and summer in the Northern Hemisphere, will not automatically result in a decrease in COVID-19 cases unless comprehensive public health measures are taken [66]. These results were confirmed by Renato H. L. Pedrosa for 50 U.S. states and 110 countries [46]. Other studies have reported conflicting results. One study reported that relative humidity was inversely related to daily deaths from COVID-19 (r = −0.32), with the largest reduction at lag 3 [67]. Yu Wu et al. collected daily data on climate, as well as the number of new COVID-19 cases and deaths, for 166 countries (excluding China) up until March 27, 2020. Their findings provide preliminary evidence that the COVID-19 pandemic could be partially mitigated by increases in both humidity and temperature [68]. Several studies have found no correlation between COVID-19 cases and humidity [60,69].

In the current study, all acute patients were confirmed by real-time PCR; however, some sub-acute patients who did not visit clinical and diagnostic centers may have been missed and, therefore, were not included in the study. Additionally, our team lacked data on socioeconomic status, healthcare access, and similar factors during the epidemic, which could not be incorporated into the analysis. These represent some limitations of our work.

## 5. Conclusion

The current study identified several environmental and climatic factors influencing the occurrence of COVID-19. Urban setting, irrigated farms, dry farms, evaporation, and altitude were the most important geoclimatic determinants of COVID-19 in the studied areas of southwest Iran. Urban life with higher human contact and surrounding farmland probably due to greater potential for dust can facilitate transmission of COVID19. Higher altitude and evaporation slightly decreased the chance of disease probably due to some physiological adaptations in people living in higher elevations and reducing transmission via virus-containing droplets. Humidity, rainfall, frequency of dusty days and thin rangeland and forest were only considered determinants when evaluated independently. Higher rainfall, frequent dusty days, and lower humidity showed a modest protective effect. Thin forests and rangelands had a positive association with disease incidence, possibly because people visited these areas more frequently to avoid urban crowds during the epidemic. These findings suggest that regions with these characteristics may exhibit higher vulnerability to viral transmission and infection. The implications of these results are significant for public health policy and urban planning. Understanding the interplay of these geographical and climatic factors can guide the development of targeted mitigation strategies, such as prioritizing vaccination campaigns in high-risk areas and implementing effective land management practices. Furthermore, these insights could be instrumental in preparing for future pandemics by fostering resilience in communities that face similar environmental challenges.
